# Long-term studies of hantavirus reservoir populations in the southwestern United States: a synthesis.

**DOI:** 10.3201/eid0501.990116

**Published:** 1999

**Authors:** J N Mills, T G Ksiazek, C J Peters, J E Childs

**Affiliations:** Centers for Disease Control and Prevention, Atlanta, Georgia 30333, USA. jum0@cdc.gov

## Abstract

A series of intensive, longitudinal, mark-recapture studies of hantavirus infection dynamics in reservoir populations in the southwestern United States indicates consistent patterns as well as important differences among sites and host-virus associations. All studies found a higher prevalence of infection in older (particularly male) mice; one study associated wounds with seropositivity. These findings are consistent with horizontal transmission and transmission through fighting between adult male rodents. Despite very low rodent densities at some sites, low-level hantavirus infection continued, perhaps because of persistent infection in a few long-lived rodents or periodic reintroduction of virus from neighboring populations. Prevalence of hantavirus antibody showed seasonal and multiyear patterns that suggested a delayed density-dependent relationship between prevalence and population density. Clear differences in population dynamics and patterns of infection among sites, sampling periods, and host species underscore the importance of replication and continuity of long-term reservoir studies. Nevertheless, the measurable associations between environmental variables, reservoir population density, rates of virus transmission, and prevalence of infection in host populations may improve our capacity to model processes influencing infection and predict increased risk for hantavirus transmission to humans.


### Long-Term Studies of Hantavirus Reservoir Populations in the Southwestern United States: A Synthesis

**James N. Mills, Thomas G. Ksiazek, C.J. Peters, and James E. Childs**
Centers for Disease Control and Prevention, Atlanta, Georgia, USA

A series of intensive, longitudinal, mark-recapture studies of hantavirus infection dynamics in reservoir populations in the southwestern United States indicates consistent patterns as well as important differences among sites and host-virus associations. All studies found a higher prevalence of infection in older (particularly male) mice; one study associated wounds with seropositivity. These findings are consistent with horizontal transmission and transmission through fighting between adult male rodents. Despite very low rodent densities at some sites, low-level hantavirus infection continued, perhaps because of persistent infection in a few long-lived rodents or periodic reintroduction of virus from neighboring populations. Prevalence of hantavirus antibody showed seasonal and multiyear patterns that suggested a delayed density-dependent relationship between prevalence and population density. Clear differences in population dynamics and patterns of infection among sites, sampling periods, and host species underscore the importance of replication and continuity of long-term reservoir studies. Nevertheless, the measurable associations between environmental variables, reservoir population density, rates of virus transmission, and prevalence of infection in host populations may improve our capacity to model processes influencing infection and predict increased risk for hantavirus transmission to humans.

A series of ongoing studies of the natural history of hantavirus-host associations in the southwestern United States was conducted by four independent investigative teams in a variety of ecosystems. The studies, which have a common experimental design, describe several patterns common to all study sites; provide insight into hantavirus maintenance in natural reservoir populations; highlight differences among geographic regions, ecosystems, and closely related host-virus associations; and illustrate that different sigmodontine rodent species (even within the genus *Peromyscus*) may respond differently to the same environmental conditions at the same site.

Sin Nombre virus (SNV), whose host is the deer mouse (*Peromyscus maniculatus*), has been responsible for most, if not all, cases of hantavirus pulmonary syndrome (HPS) in the southwestern United States since 1993. Deer mouse population density and prevalence of SNV infection in deer mouse populations in the arid Southwest have declined sharply since the high levels documented in 1993 (1-3). Nevertheless, moderate population densities of deer mice persisting at the higher altitude web trapping sites in Colorado provided an opportunity to look at the natural history of this species over a wide range of conditions (Calisher et al., this issue, pp. 126-134).

The high prevalence of SNV-reactive antibody in brush mouse (*P. boylii*) populations observed during these and previous studies in the Southwest (2) led to the investigation and identification of a distinct hantavirus carried by brush mice (S. Nichol and A. Johnson, unpub. data). Before these studies were undertaken, it was not known whether antibody in brush mice represented spillover of SNV from the deer mouse reservoir (as may have been the case during the initial 1993 outbreak), unusual maintenance of the same hantavirus by two species of rodents, or (as molecular evidence now indicates) another example of cospeciation leading to a unique hantavirus maintained in a single rodent species. Although the status of the

Address for correspondence: James N. Mills, Centers for Disease Control and Prevention, Mailstop G14, 1600 Clifton Road, N.E., Atlanta, GA 30333, USA; fax: 404-639-1118; e-mail: jum0@cdc.gov.

virus associated with *P. boylii* as a human pathogen is unknown, *P. boylii* is a common species in the Southwest, and its population density fluctuates dramatically with environmental conditions. Data on the brush mouse host-virus association can contribute to our understanding of hantavirus reservoir ecology.

We summarize major conclusions from the first 3 years of hantavirus reservoir studies in the southwestern United States, examine consistent patterns and salient differences, and discuss the implications of the studies for understanding reservoir host ecology.

### Temporal Patterns

### In Host Populations

#### Multiyear Patterns

In most rodent communities examined, sampling methods using trapping webs demonstrated periodic fluctuations in population densities; many populations were simultaneously adversely affected by periods of low rainfall. In the southwestern United States, unusually dry conditions directly followed the wet, exceptionally favorable conditions believed to have contributed to the increase in rodent numbers and the HPS outbreak of 1993-94.

Specific habitat characteristics critical to some hantavirus reservoir species are the availability of thick chaparral cover for brush mice (Abbott et al., this issue, pp. 102-112) and food supply, including acorns and other fruits and seeds (this issue, Calisher et al. pp. 126-134 and Abbott et al., pp. 102-112). As il lustrated by the different effects of drought on pinyon mice (*P. truei*) and brush mice in northern Arizona (Abbott et al., this issue, pp. 102-112), the response of a rodent population to ecologic conditions depends on its specific requirements for food, water, and habitat. A complete understanding of the ecologic requirements and adaptability of each reservoir species is required before the species' response to specific environmental conditions and its potential contribution to future outbreaks of HPS can be predicted.

Many species of murid rodents typically exhibit year-to-year fluctuations in population density. The Arvicolinae are a panarctic group containing several species that undergo fairly regular population cycles with a 3- to 4-year periodicity. Periodic fluctuations in populations of the bank vole (*Clethrionomys glareolus*), an arvicoline rodent and the reservoir for Puumala virus (a hantavirus that causes a mild form of hemorrhagic fever with renal syndrome [nephropathia epidemica] in Europe), have been related to nephropathia epidemica incidence in Sweden (4). The causes of these population cycles in arvicoline rodents are not well understood (5,6). All viruses known to cause HPS are carried by rodents of the New World murid subfamily Sigmodontinae. Although sigmodontine rodent populations do not fluctuate on a regular, cyclic basis, periodic, sometimes dramatic increases ("irruptions") occur in population density; these increases may be tied to unusual climatic events that result in highly favorable (if temporary) conditions for nutrition and reproduction. Such an increase involving deer mouse populations may have been associated with the first recognized outbreak of HPS in the southwestern United States in 1993 (7). Understanding the factors that control or influence the population dynamics of sigmodontine reservoir species is central to understanding the epidemiology of HPS.

#### Seasonal Patterns

In addition to the overall year-to-year trends in rodent population dynamics, some populations demonstrated seasonal patterns that varied by ecosystem: the size of brush mice and deer mice populations at desert grassland sites peaked in winter and waned in midsummer, while at high altitude sites in Colorado, it was generally highest in the fall. Models of disease risk to humans must consider altitude and biome, as well as regional weather patterns.

### In Prevalence of Infection

#### Assumptions Concerning Antibody Analyses

In these studies, we assume that antibody-positive hosts (*P. maniculatus*, *P. boylii,* and *Reithrodontomys megalotis*) are chronically infected and infectious. Studies of other specific hantavirus-host associations, including Hantaan virus in *Apodemus agrarius* (8), Puumala virus in *C. glareolus* (9,10), and Black Creek Canal virus in *Sigmodon hispidus* (11), show a similar pattern: infection is followed by a brief period of viremia and then by the development of antibody and clearing of virus from blood. Nevertheless, in spite of the continuous presence of circulating antibody, high-titer virus could be isolated from organs, and infectious virus was shed persistently or sporadically in urine, feces, and saliva for extended periods, probably the life of the host. The quantities of virus shed may be greatest during the early phases (2 to 4 weeks postinoculation) of infection (8). In a field study of SNV, 97% of antibody-positive *P. maniculatus* had viral RNA in organ tissue (1), which implies a similar pattern of chronic infection for the deer mouse–SNV association.

These studies confirm the cited laboratory investigations demonstrating the maintenance of antibody for the expected life span (approximately 1 year or less) of the host. Antibody was detected by enzyme-linked immunosorbent assay (ELISA) for up to 16 months in individual rodents, and no mice reverted from antibody-positive to antibody-negative. Nevertheless, loss of antibody may be observed in animals born with transient maternal antibody (2,12).

Finally, these studies used antibody as the only marker of infection. Mice sampled between infection and development of detectable antibody (probably 3 to 4 weeks [11]) are not recognized as infected; these may represent 2% to 7% of animals sampled (13,14). In one study (1), 55% of seronegative animals had viral RNA in blood samples; however, this study was unusual—it was conducted during epizootic conditions, which presumably involved very high rates of transmission in the reservoir population. In addition, the use of a heterologous antigen (Prospect Hill virus) to detect SNV antibody in the ELISA format may have slightly decreased the sensitivity of serologic tests. Thus, although the actual correction factor is imprecisely known and probably variable, the seroprevalence provided in these reports underestimate the true prevalence of infection in host populations.

### Multiyear Patterns

These studies support previous investigations (2,12,15) demonstrating that rodents do not acquire hantavirus infection vertically but instead become infected (and presumably infectious) and develop antibody in an age- or size-related manner. Infection appears associated with life history and behavioral events surrounding the maturation of animals into sexually mature adults. Given the horizontal transmission of hantavirus within reservoir populations, increasing population densities should result in increased rodent-to-rodent contact, opportunities for virus transmission (to susceptible mice), and overall incidence and cumulative prevalence of infection within host populations. Such findings would be consistent with the mass action principle of disease transmission, which assumes that transmission is a function of density (16). Nevertheless, clear evidence of increased population densities leading to increased prevalence of infection in hantavirus host populations is lacking (2,14,17,18). Indeed, many datasets, such as that presented by Abbott et al. (this issue, pp. 102-112), show an inverse relationship between population density and antibody prevalence over time.

These same data, however, can provide insights into the interaction of temporal patterns of reproduction, changing population age structure, and virus transmission. For example, the successful breeding seasons for brush mice in northern Arizona (spring through fall 1995 and spring 1996) resulted in a population with a high proportion of juvenile and young mice not yet infected (as evidenced by antibody). Increasing population density resulted in increasing incidence of virus transmission (as evidenced by the high rate of seroconversion during this period), but the overall antibody prevalence in the population was continuously diluted and offset by the addition of uninfected juvenile mice. By summer 1996, however, local environmental conditions caused breeding to end and production of young to subsequently decline. Until noninfected susceptible young mice began to enter the population again during the summer of 1997, the population consisted of older residents that, by virtue of their age and cumulative life experiences, were commonly infected with hantavirus. Thus, the relatively high prevalence of infection during this period reflects the high rate of transmission during the previous fall, the subsequent decline in new births, and the resultant older age structure and accumulated life experience of the population.

### Seasonal Patterns

The dynamics of changing population structure and virus transmission may also result in predictable seasonal patterns in the prevalence of infection. In strongly seasonal climates, the interplay of host demography and horizontal virus transmission may result in a strongly seasonal alternation of peaks in population density and prevalence of infection. As an example of other hantavirus-host associations, in Sweden, bank vole population density was highest in the fall, but the prevalence of Puumala virus antibody was highest in the spring and correlated with vole density the previous fall and spring (4).

This delayed density-dependent prevalence of infection occurs in other reservoir populations in strongly seasonal environments, such as the high-altitude grids near Fort Lewis, Colorado (Calisher et al., this issue, pp. 126-134). Every year, except 1994, when populations may have been recovering from El Niño southern oscillation conditions and thus showed an atypical pattern, population density of *P. maniculatus* was lowest in the early spring (presumably because of the high number of winter deaths) and increased throughout the breeding season, into summer and fall. Furthermore, in 1995 and 1997 (no antibody-positive animals were captured in 1996), antibody prevalence was highest in the early spring and lower in the fall; this pattern could be the result of reproduction resulting in highest population density in the fall but with the juvenile dilution effect, which leads to low antibody prevalence. The spring population, consisting largely of overwintering adult mice, reflects the relatively high antibody prevalence expected in older animals. The high prevalence in spring presumably reflects virus transmission in the high density population of the previous autumn.

A study of hantavirus in rodent communities in Argentina provides additional evidence for the broad applicability of this pattern in temperate ecosystems. Several hantavirus reservoir species on the central Argentine pampa displayed the same spring-fall alternation of peaks in population density and antibody prevalence (19). Thus, the temporal asynchrony between reservoir population density and prevalence of infection on both the year-to-year and within-year scales can be explained by the interaction of seasonal changes in population structure and horizontal transmission of virus.

The explanations for this pattern suggest three corollary hypotheses: virus "overwinters" in temperate rodent communities as persistent infections in older adult animals, which serve as a reservoir for reintroducing virus into susceptible young animals in the spring; spring antibody prevalence is a function of the population density (infectious and susceptible) the year before (the high fall population densities and higher spring antibody prevalence at the Colorado trapping webs in spring 1995 provide tentative support for this hypothesis); and deviations from typical environmental conditions alter the pattern of infection in potentially predictable directions. For example, a mild winter might prolong the period of reproduction and transmission, simultaneously increasing population densities and improving overwinter survival. Such conditions might result in an atypically high prevalence of infection, as well as a higher-than-usual population base in the spring. Such a pattern might help account for the conditions of high population densities and high prevalence of infection during the initial phases of the HPS outbreak in the southwestern United States in the spring of 1993 (1). Expected changes in population density, antibody prevalence, and population age structure over a hypothetical multiyear cycle are shown in the Figure.

Prevalence of infection in reservoir populations, however, is only one of several factors that may be useful in predicting risk for human disease. The highest absolute numbers of infected rodents (but not prevalence) coincided with high population density (Abbott et al., this issue, pp. 102-112); thus, all other factors being equal, the highest risk for human contact with infected rodents would be during the period of highest rodent population density. Factors of the host-virus interaction (e.g., time course of infection and periods of maximum virus shedding), rodent behavior (e.g., entering human habitations), and human behavior (e.g., planting or harvesting in the spring and fall and opening and cleaning rodent-infested sheds or cabins in the spring) interact to modify specific temporal risk patterns.

### In Virus Transmission

The two reports that documented a high incidence of infections as evidenced by first acquisition of antibody (this issue, Calisher et al., pp. 126-134 and Abbott et al., pp. 102-112) provide evidence for seasonal patterns in transmission—one (Abbott et al., this issue, pp. 102-112) clearly documented that the highest rates of seroconversion corresponded with highest population density. The apparently different seasonal patterns of seroconversion of male and female animals in Colorado were unexpected (Calisher et al., this issue, pp. 126-134). The Colorado study suggests that winter transmission of virus occurs during communal "nesting." This may help explain why brush mice, living in desert and brushland habitats with milder winters, have a higher ratio of male to female antibody-positive mice. Virus transmission among brush mice may be more restricted to aggressive encounters, which would favor male infection; virus transmission among deer mice at high altitudes might also include opportunities for transmission during communal nesting (e.g., by aerosol or mutual grooming),

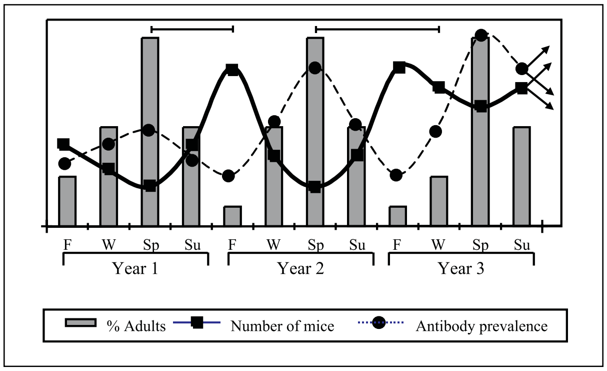

Figure. A hypothetical schematic of seasonal changes in hantavirus prevalence, rodent host population density, and population age structure.In the first autumn, after a normal breeding season, the high density population consists primarily of young not exposed to virus or recently exposed before development of antibody. Because of deaths in winter, populations decrease to a spring nadir. However, antibody prevalence is high in this population of overwintered adults exposed during the previous breeding season or during winter communal huddling. Unusually favorable conditions during the following spring and summer (first horizontal bar) result in higher population density the second fall, and the increased influx of young of the year (juvenile dilution effect) results in an even lower antibody prevalence than the previous fall. A typical winter results in a high number of winter deaths and a typical low density spring population, but seroprevalence the second spring is higher because of increased opportunities for transmission events in the high density population of the previous fall. The second extended favorable season (second horizontal bar) leads again to high population density and low seroprevalence in autumn. The reservoir population of the third spring demonstrates high antibody prevalence, because of high rates of exposure during the "crowded" conditions of the previous fall, and unusually high population density, because of extended breeding and high overwinter survival. Depending on environmental conditions, the population may abruptly "crash" (if it has exceeded the carrying capacity of the environment) or might continue to increase (e.g., if growing conditions the previous spring and summer resulted in abundant food supplies, such as a mast crop of acorns or pinyon nuts).

which could diminish differences in transmission ratios between male and female mice.

These studies indicate that hantavirus infection was resilient in the face of population fluctuations. Even when rodent populations were very low, some foci of infection were apparently sustained, presumably through persistent infection in a few long-lived residents (Abbott et al., this issue, pp. 102-112). However, other data indicate that infection may disappear completely from a population during periods of low density, only to reappear sporadically in a few infected mice (Kuenzi et al., this issue, pp. 113-117). The latter phenomenon could indicate that low levels of infection were continuously present in the population, but the sampling method was not sensitive enough to detect it; or it may indicate that virus periodically becomes extinct in semi-isolated populations that have declined in numbers. Virus might be reintroduced into a population through contact with, or dispersal of infected rodents from, adjacent populations, which suggests that reservoir species should be considered metapopulations in maintaining hantavirus infection.

### Spatial Patterns

Several reports in this series provide evidence for spatial restrictions in the geographic distribution of rodent reservoir populations, as well as for focality of infection within populations. Although focality has been observed on a regional scale (2), the data by Kuenzi et al. (this issue, pp. 113-117) and Abbott et al. (this issue, pp. 102-112) demonstrated distinct "islands" of hantavirus infection apparently associated with preferred microhabitat for brush mice on web trapping sites, a pattern reminiscent of the concept of natural "nidality" of zoonotic disease as expounded by Pavlovsky (20). Characterizing preferred habitat types may help identify areas at increased risk for virus transmission to humans. However, these pockets of reservoir activity become blurred during periods of high reservoir population density (Abbott et al., this issue, pp. 102-112). Not only would it be more difficult to identify foci that pose a high risk for virus infection during reservoir population irruptions, but also the increased movement of individual rodents may lead to transfer of virus among previously distinct subpopulations, increasing the overall risk for human exposure.

### Characteristics of Infected Populations

At all sites and for both brush and deer mice, infected animals were more frequently older males. These data are consistent with horizontal transmission of infection and suggest that the (or a) specific mode of transmission involves male more frequently than female animals. An alternative hypothesis, that males live longer than females (which would lead to greater cumulative probability of infection), is inadequate (Kuenzi et al., this issue, pp. 113-117); therefore, behavioral differences (e.g., greater home range, increased aggression, bites, wounding) may be the most likely explanation. Indeed male murid rodents may be more likely to have scars or wounds (indicators of aggressive encounters) than female rodents (19,21); the presence of scars has been associated with increased prevalence of infection for hantaviruses (12;19; Calisher et al., this issue, pp. 126-134).

Nevertheless, patterns of antibody prevalence differed distinctly by site and species. For instance, the male bias among infected animals was much greater for brush mice in Arizona (85% to 90% of infected animals were male [this issue, Abbott et al., pp. 102-112 and Kuenzi et al., pp. 113-117]) than for deer mice in Colorado (approximately 60% [Calisher et al., this issue, pp. 126-134]). These differences presumably relate to intersite or interspecies differences in social structure or behavior that influence hantavirus transmission. Are female deer mice more likely to fight or otherwise interact than female brush mice? Does winter communal nesting facilitate transmission among both male and female deer mice and not brush mice? Could venereal transmission be involved for deer mice? Some of these questions can be addressed only by continued data collection at the long-term trapping webs. For instance, collection and analysis of data concerning wounding and scars should document the relative frequency of aggressive encounters among males and females of all species. As demonstrated by Abbott et al. (this issue, pp. 102-112), comparing data on interactions of same sex and opposite sex mice involved in dual captures may also yield insights. In fact, preliminary analysis of scar frequency by Calisher et al. (this issue, pp. 126-134) indicates that male deer mice in Colorado may not experience more aggressive encounters than female mice; dual-capture results by Abbott et al. (this issue, pp. 102-112) show that male-male interactions among brush mice can be considerably more aggressive than female-female interactions. If communal nesting increases viral transmission between deer mice, the pattern of antibody prevalence among male and female mice may differ for deer mice captured at trapping webs in lower altitude sites in New Mexico and eastern Colorado. These data are being collected. Venereal transmission, which would be difficult to address in field studies, will require parallel studies in the laboratory.

### Comparison of SNV Prevalence with Prevalence of Other Rodent-Borne Agents

The prevalence of infection with hantavirus shown by these studies is 0% to approximately 25%. Even under conditions of high rodent density in the areas of human disease outbreaks, the prevalence of SNV infection in deer mice reached only 30% (1). The high rate of population turnover and relatively short life span of most sigmodontine hosts results in populations frequently dominated by young mice not yet infected with hantavirus; the delay between infection and development of antibody further decreases the apparent prevalence of infection. However, when data are stratified by age and sex, antibody prevalence can be high. For example, 90% of male Norway rats >500 g in Baltimore, Maryland, had antibody to Seoul virus (15), and 88% of male cotton rats >200 g in southern Florida had antibody reactive with SNV (12). These prevalences are comparable to the highest prevalences for agents reported to be vertically transmitted such as some arenaviruses including Lassa (22) and lymphocytic choriomeningitis viruses (23).

### The Future

Preliminary results from these studies indicate that some patterns, such as age- and male-associated infection, are clear. Nevertheless, upon closer inspection, the patterns differ between sites and species. Reservoir studies at one site, in one ecosystem, during 1 year, or of one host-virus system cannot provide the data necessary to piece together the natural history of hantavirus infection in North American reservoirs. Environmental conditions cannot be controlled in the field; therefore, adequate replication of field studies across time, space, and host-virus systems is critical. Although extensive, the studies reported in this series are preliminary. Three years is a very brief period for detecting effects due to environmental changes (e.g., weather and landscape) and for detecting the impact of extremely rare events (e.g., a 20-year flood). The conditions that lead to rodent population irruptions may be infrequent, and there may be thresholds for either environmental conditions or population densities that lead to the increased numbers of infected rodents that are indicators of risk of virus transmission to humans. The ultimate usefulness of these studies depends upon their long-term maintenance.

The methods used in these studies appear sensitive enough to detect changes in reservoir populations associated with increased virus transmission. The sampling methods did not significantly increase deaths among study animals (this issue, Calisher et al., pp. 126-134 and Abbott et al., pp. 94-104; 24;25). The few unavoidable deaths associated with periodic bleeding of animals and mark-recapture studies do not affect most population estimates; the statistical analyses are sufficiently sensitive to detect intersite and temporal differences in population densities (Parmenter et al., this issue, pp. 118-125).

But can these studies provide early warning of conditions that predate and predict an increase in virus transmission and HPS? Data from the last few months of the study period show an abrupt increase in the population density of some reservoir species that coincides with habitat improvements, most likely resulting from increases in rainfall associated with an El Niño southern oscillation event beginning in 1997. The current environmental changes may provide a rare opportunity to document the weather and ecologic conditions associated with demographic changes in reservoir host populations that increase risk for virus transmission to human populations. Recent increases in reservoir populations have been associated with increased numbers of HPS cases in the southwestern United States. As of August 1998, approximately 14 cases have been reported in Arizona, Colorado, New Mexico, and Utah, in comparison to 2, 2, and 4, for the same period in 1995, 1996, and 1997 (A. Khan, unpub. data). The qualitative and quantitative data on reservoir populations and environmental variables collected during this period may also provide the necessary habitat-specific correlations so that satellite images can be related to specific environmental clues. When these links are established, the wide coverage offered by remote sensing platforms may provide the capability to predict increased risk in areas without direct reservoir monitoring.

Even though the variety of ecosystems and host-virus systems included in these studies may lead to models with broad applicability, they still represent a relatively small geographic area and a small percentage of the known hantavirus-host associations in the world. Similar studies in other areas of the United States provide comparisons (17), but similar studies of other sigmodontine reservoirs in South America and arvicoline and murine reservoirs in Europe and Asia are needed.

Finally, the value of these longitudinal studies will increase when these data are integrated with data from complementary field and laboratory studies. These mark-recapture studies are restricted to wild populations in natural environments, while most human cases of HPS are acquired in the peridomestic environment. Although the dynamics of natural populations ultimately influence the density and behavior of peridomestic deer mice, for example, the specific factors of human and rodent behavior that lead to peridomestic exposure can be elucidated only through studies in the specific environment of exposure.

The presence of IgG antibody reactive with SNV antigen is used as the marker of infection in these studies. Given the pattern of chronic infection and long-term shedding of virus in hantavirus-host associations (8,9,11), antibody is probably a good marker. Nevertheless, the specific dynamics and timing of infection, antibody development, and timing of maximum viral shedding are unknown for most hantavirus reservoir species. These data must be provided by controlled laboratory studies using artificially infected animals and (because laboratory infections may not always mimic natural infections [26]) field studies involving naturally infected animals. Natural or manipulative field studies might use captive populations in seminatural enclosures or excretory products collected (by use of metabolic chambers) from wild-caught animals in mark-recapture studies; the success of these studies will depend on the development of assays for infectious virus.

### Acknowledgment

Barbara Ellis provided the graphics and helpful suggestions that improved the manuscript.

Dr. Mills is chief of the Medical Ecology Unit, Special Pathogens Branch, Division of Viral and Rickettsial Diseases, CDC. His research interests include zoonotic diseases, specifically host-pathogen evolution and interactions.

### References

1. Childs JE, Ksiazek TG, Spiropoulou CF, Krebs JW, Morzunov S, Maupin GO, et al. Serologic and genetic identification of *Peromyscus maniculatus* as the primary rodent reservoir for a new hantavirus in the southwestern United States. J Infect Dis 1994;169:1271-80.
2. Mills JN, Ksiazek TG, Ellis BA, Rollin PE, Nichol ST, Yates TL, et al. Patterns of association with host and habitat: antibody reactive with Sin Nombre virus in small mammals in the major biotic communities of the southwestern United States. Am J Trop Med Hyg 1997;56:273-84.
3. Engelthaler DM, Levy CE, Fink M, Tanda D, Davis T. Short report: decrease in seroprevalence of antibodies to hantavirus in rodents from 1993-1994 hantavirus pulmonary syndrome case sites. Am J Trop Med Hyg 1998;58:737-8.
4. Niklasson B, Hornfeldt B, Lundkvist A, Bjorsten S, LeDuc J. Temporal dynamics of Puumala virus antibody prevalence in voles and of nephropathia epidemica incidence in humans. Am J Trop Med Hyg 1995;53:134-40.
5. Krebs CJ, Myers JH. Population cycles in small mammals. Advances in Ecological Research 1974;8:267-399.
6. Hornfeldt B. Delayed density dependence as a determinant of vole cycles. Ecology 1994;75:791-806.
7. Parmenter RR, Brunt JW, Moore DI, Ernest S. The hantavirus epidemic in the southwest: rodent population dynamics and the implications for transmission of hantavirus-associated adult respiratory distress syndrome (HARDS) in the four corners region. University of New Mexico. Sevilleta LTER Publication. No. 41:1993.
8. Lee HW, Lee PW, Baek LJ, Song CK, Seong IW. Intraspecific transmission of Hantaan virus, etiologic agent of Korean hemorrhagic fever, in the rodent *Apodemus agrarius*. Am J Trop Med Hyg 1981;30:1106-12.
9. Yanagihara R, Amyx HL, Gajdusek DC. Experimental infection with Puumala virus, the etiologic agent of nephropathia epidemica, in bank voles (*Clethrionomys glareolus*). J Virol 1985;55:34-8.
10. Bogdanova SB, Gavrilovskaya IN, Boyko VA, Prokhorova NA, Linev MB, Apekina NS, et al. Persistent infection caused by hemorrhagic fever with renal syndrome in red mice (*Clethrionomys glareolus*), natural hosts for the virus (in Russian, translated by SCITRAN). Mikrobiol Zh 1987;49:99-106.
11. Hutchinson KL, Rollin PE, Peters CJ. Pathogenesis of a North American hantavirus, Black Creek Canal virus, in experimentally infected *Sigmodon hispidus*. Am J Trop Med Hyg 1998;59:58-65.
12. Glass GE, Livingstone W, Mills JN, Hlady WJ, Fine JB, Rollin PE, et al. Black Creek Canal virus infection in *Sigmodon hispidus* in southern Florida. Am J Trop Med Hyg 1998;59:699-703.
13. Otteson EW, Riolo J, Rowe JE, Nichol ST, Ksiazek TG, Rollin PE, et al. Occurrence of hantavirus within the rodent population of northeastern California and Nevada. Am J Trop Med Hyg 1996;54:127-33.
14. Boone JD, Otteson EW, McGwire KC, Villard P, Rowe JE, St Jeor SC. Ecology and demographics of hantavirus infections in rodent populations in the Walker River basin of Nevada and California. Am J Trop Med Hyg 1998;58:445-51.
15. Childs JE, Korch GW, Glass GE, LeDuc JW, Shah KV. Epizootiology of hantavirus infections in Baltimore: isolation of a virus from Norway rats, and characteristics of infected rat populations. Am J Epidemiol 1987;126:55-68.
16. Dobson AP, Hudson PJ. Microparasites: observed patterns in wild animal populations. In: Grenfell BT, Dobson AP, editors. Ecology of infectious diseases in natural populations. Cambridge: Cambridge University Press; 1995. p. 52-89.
17. Douglass RJ, Van Horn R, Coffin K, Zanto SN. Hantavirus in Montana deer mouse populations: preliminary results. J Wildl Dis 1996;32:527-30.
18. Bond CW, Irvine B, Alterson HM, Van Horn R, Douglass RJ. Longitudinal incidence of hantavirus infection in deer mice. Fourth International Conference on HFRS and Hantaviruses, Mar 5-7 1998, Atlanta, Georgia. Centers for Disease Control and Prevention, Atlanta, GA.
19. Mills JN, Schmidt K, Ellis BA, Ksiazek TG. Epizootiology of hantaviruses in sigmodontine rodents on the pampa of central Argentina. Euro-American Mammal Congress, Universidad de Santiago de Compostela, Spain, Jul 19-24, 1998. Published by Universidad de Santiago de Compostela, Santiago de Compostela, Spain.
20. Pavlovsky EN. Natural nidality of transmissible diseases. Urbana: University of Illinois Press; 1966. p. 1-261.
21. Mills JN, Ellis BA, McKee KT, Calderón GE, Maiztegui JI, Nelson GO, et al. A longitudinal study of Junín virus activity in the rodent reservoir of Argentine hemorrhagic fever. Am J Trop Med Hyg 1992;47:749-63.
22. McCormick JB, Webb PA, Krebs JW, Johnson KM, Smith ES. A prospective study of the epidemiology and ecology of Lassa Fever. J Infect Dis 1987;155:437-44.
23. Skinner HH, Knight EH, Grove R. Murine lymphocytic choriomeningitis: the history of a natural cross-infection from wild to laboratory mice. Lab Anim 1977;11:219-22.
24. Parmenter CA, Yates TL, Parmenter RR, Mills JN, Childs JE, Campbell ML, et al. Small mammal survival and trapability in mark-recapture monitoring programs for hantavirus. J Wildl Dis 1998;34:1-12.
25. Swann DE, Kuenzi AJ, Morrison ML, DeStefano S. Effects of sampling blood on survival of small mammals. Journal of Mammalogy 1997;78:908-13.
26. Mills JN, Childs JE. Ecological studies of rodent reservoirs: their relevance for human health. Emerg Infect Dis 1998;4:
